# WHAT MATTERS TO CHINESE AND KOREAN DEMENTIA CAREGIVERS: INFORMING THE CULTURAL ADAPTATION OF THE NYUCI-ES PROGRAM

**DOI:** 10.1093/geroni/igad104.0483

**Published:** 2023-12-21

**Authors:** Jing Wang, Bei Wu, Min Kyoung Johnson, I tek Leong, Yaolin Pei, Cynthia Epstein, Soyeon Cho, Mary Mittelman

**Affiliations:** University of New Hampshire, Durham, New Hampshire, United States; New York University, New York City, New York, United States; New York University Rory Meyers College Of Nursing, New York City, New York, United States; New York University Rory Meyers College Of Nursing, New York City, New York, United States; New York University, New York City, New York, United States; NYU Grossman School of Medicine, New York City, New York, United States; City University of New York, New York City, New York, United States; NYU Langone HealthNYU Grossman School of Medicine, New York City, New York, United States

## Abstract

Family caregivers of persons with dementia (PWD) undertake an extensive amount of care and many are challenged by heavy caregiving burden and their own health problems. The New York University Caregiver Intervention (NYUCI) program has demonstrated effectiveness in multiple studies among largely Caucasian samples in improving the wellbeing of caregivers of PWD by decreasing their levels of depression and stress and improving self-reported health. As two large Asian immigrant population in the U.S., Korean and Chinese American caregivers of PWD have unique challenges impacted by their cultural background. However, there are limited studies on what can be done to support them in a culturally sensitive way. The current study aims to inform the cultural adaptation of the program for Chinese and Korean caregivers with multiple chronic conditions by exploring their perspectives to understand their unique challenges, unmet needs, and the best ways to support them in caregiving and engage them in selfcare. We interviewed 14 Chinese and 11 Korean caregivers living in the New York Metropolitan Area. We compared themes mentioned by both groups and found that cultural beliefs profoundly impact their perceptions of dementia and caregiving, their way of utilizing social and family support, and understanding of self-care and counselling services. Caregivers are facing and addressing new challenges during and after the Pandemic, providing insights into how we can use technology and telehealth to assist them in caregiving and coordinating support from others. Our findings provided implications to culturally adapt the NYUCI program to culturally diverse populations.

